# Diagnostic performance of deep learning and computational fluid dynamics-based instantaneous wave-free ratio derived from computed tomography angiography

**DOI:** 10.1186/s12872-022-02469-0

**Published:** 2022-02-05

**Authors:** Jingyuan Zhang, Kun Xu, Yumeng Hu, Lin Yang, Xiaochang Leng, Hongfeng Jin, Yiming Tang, Xiaowei Liu, Chen Ye, Yitao Guo, Lei Wang, Jianjun Zhang, Yue Feng, Caiyun Mou, Lijiang Tang, Jianping Xiang, Changqing Du

**Affiliations:** 1 Department of Medicine, The Second College of Clinical Medicine, Zhejiang Chinese Medical University, Hangzhou, China; 2 ArteryFlow Technology Co., Ltd., 459 Qianmo Road, Hangzhou, 310051 China; 3 Department of Geriatrics, The First Affiliated Hospital, Zhejiang University School of Medicine, Hangzhou, China; 4 Department of Cardiology, Zhejiang Hospital, Hangzhou, 310013 China; 5 Department of Radiology, Zhejiang Hospital, Hangzhou, China

**Keywords:** Coronary artery disease, Fractional flow reserve (FFR), AccuiFRct, Instantaneous wave-free ratio (iFR), Physiology

## Abstract

**Background and objectives:**

Both fractional flow reserve (FFR) and instantaneous wave-free ratio (iFR) are widely used to evaluate ischemia-causing coronary lesions. A new method of CT-iFR, namely AccuiFRct, for calculating iFR based on deep learning and computational fluid dynamics (CFD) using coronary computed tomography angiography (CCTA) has been proposed. In this study, the diagnostic performance of AccuiFRct was thoroughly assessed using iFR as the reference standard.

**Methods:**

Data of a total of 36 consecutive patients with 36 vessels from a single-center who underwent CCTA, invasive FFR, and iFR were retrospectively analyzed. The CT-derived iFR values were computed using a novel deep learning and CFD-based model.

**Results:**

Mean values of FFR and iFR were 0.80 ± 0.10 and 0.91 ± 0.06, respectively. AccuiFRct was well correlated with FFR and iFR (correlation coefficients, 0.67 and 0.68, respectively). The diagnostic accuracy, sensitivity, specificity, positive predictive value, and negative predictive value of AccuiFRct ≤ 0.89 for predicting FFR ≤ 0.80 were 78%, 73%, 81%, 73%, and 81%, respectively. Those of AccuiFRct ≤ 0.89 for predicting iFR ≤ 0.89 were 81%, 73%, 86%, 79%, and 82%, respectively. AccuiFRct showed a similar discriminant function when FFR or iFR were used as reference standards.

**Conclusion:**

AccuiFRct could be a promising noninvasive tool for detection of ischemia-causing coronary stenosis, as well as facilitating in making reliable clinical decisions.

## Introduction

Coronary computed tomography angiography (CCTA) has been developed as a relatively simple, accurate, and low-risk noninvasive tool to assess the severity of anatomical coronary artery stenosis [1]. VERDICT study showed that CCTA was comparable to invasive coronary angiography (ICA) in assessing long-term risk in patients with non-ST-segment elevation acute coronary syndromes [2]. However, anatomical evaluation alone cannot accurately assess the major adverse cardiac events (MACE) and guide treatment strategies. Anatomical assessment combined with functional assessment can reliably evaluate functional significance of coronary artery disease (CAD). Functional assessment of coronary artery stenosis has shown to play an important role in interventional cardiology. To date, more than 10 indices have been presented for coronary physiological assessment [3]. Fractional flow reserve (FFR) is recognized as a gold standard for the assessment of physiological significance of a coronary stenosis [4]. Instantaneous wave-free ratio (iFR) was proven to be non-inferior to FFR, which can be measured without administration of vasodilators [5, 6]. DEFINE-FLAIR study and SWEDEHEART study reported that iFR-guided coronary revascularization demonstrated a prognostic value similar to FFR-guided revascularization in terms of the risk of MACE [7, 8]. The European (Class I, Level of Evidence: A) and American guidelines (Class IIa, Level of Evidence: A) have concentrated on the role of FFR or iFR in defining the functional significance of intermediate epicardial coronary stenosis [9, 10]. However, as FFR and iFR are invasive methods, the adoption rates of both methods are low in clinical practice [11]. Therefore, the exploration of non- or less invasive methods to evaluate functional ischemia is critical.

Noninvasive tools compared with FFR have been introduced for the functional assessment of coronary artery stenosis. FFR derived from CCTA (FFR_CT_) has been used in the comprehensive assessment of the anatomical and physiological functions of CAD. DISCOVER-FLOW study and NXT study showed that FFR_CT_ is highly correlated with invasive FFR [12, 13]. Several clinical trials and meta-analyses have shown a significant improvement in diagnostic accuracy compared with CCTA alone [14, 15]. Furthermore, FFR_CT_-guided patient management reduce costs and improve clinical outcomes [16, 17]. These noninvasive morphofunctional evaluation methods could reduce complications and spare procedure time and cost. However, during the computation of FFR_CT_, the boundary conditions would be converted from baseline to hyperemic to simulate the drug-induced hyperemia and to match the clinical meaning of FFR, in which this inconsistency of physiological state during computation and imaging may lead to wrong results. Thus, a CT-derived noninvasive morphofunctional approach that mimics the true physiological state of a patient during CCTA scan is worthy of further investigation.

Recently, computational methods to derive iFR from CCTA (iFR_CT_) have also shown a promising diagnostic performance in assessing functional coronary stenoses, as judged by FFR [18, 19]. However, the diagnostic performance has not been thoroughly evaluated using iFR as the reference standard. Additionally, the complexity of establishing accurate personalized coronary artery anatomic models has increased the calculation time of iFR_CT_ [18, 19]. In order to improve the accuracy and rapidity of the calculation, a novel calculation method, for calculating iFR from CCTA, based on artificial intelligence and computational fluid dynamics (CFD), including individual physiological parameters, was developed (AccuiFRct) in the present research, and then, it was attempted to evaluate the diagnostic performance and agreement of the optimized AccuiFRct algorithm using iFR as the reference standard.

## Methods

### Study design and study population

This was a retrospective, observational, single-center study. Clinical data of patients with stable CAD admitted to Zhejiang Hospital (Hangzhou, China) from August 2018 to August 2020 were collected and retrospectively analyzed. Patients who had undergone CCTA and invasive physiological measurements both FFR and iFR within 2 months were included. Inclusion criterion was as follows: patients with ≥ 1 coronary artery with 30–90% (visual estimation) stenosis from angiography. Exclusion criteria were as follows: left ventricular ejection fraction (LVEF) ≤ 35%, patients with a history of undergoing coronary artery bypass surgery or stenting for the target vessels, bifurcation stenosis, chronic total occlusion, or low-quality CCTA images. The study was conducted in accordance with the Declaration of Helsinki (as revised in 2013). The study protocol was approved by the institutional review board (IRB) (IRB number: 2020-104K). Informed consent was waived because of the retrospective nature of the study.

### Image acquisition and data analysis

CCTA was performed with a dual-source CT scanner (Siemens Healthineers, Erlangen, Germany). During the scanning, 50–70 mL contrast agent (Iopromide, 370 mg I/mL; Bayer, Leverkusen, Germany) was administrated through intravenous injection (4.5 mL/s), followed by rinsing with 40 mL saline. The retrospective electrocardiography (ECG)-gated spiral scanning was used. Scanning parameters included: detector collimation (32 × 0.6 mm) combined with z-flying focal spot technology, gantry rotation time of 330 ms, tube voltage of 120 kVp, tube current of 400 mAs, and a craniocaudal scan direction. The CCTA data were proceeded in the Central AccuiFRct Core Laboratory of ArteryFlow Technology Co., Ltd. (Hangzhou, China) for subsequent AccuiFRct calculation and analysis.

### ICA and measurement of physiological indices

ICA was performed according to standard protocols. All coronary physiological measurements were carried out after ICA. A coronary functional evaluation system and the Volcano PrimeWire PRESTIGE Plus Pressure Guide Wire (Philips Healthcare, Amsterdam, the Netherlands) were used for the physiological assessment of the target vessels. The 6F guiding catheter was utilized to measure intracoronary pressure. The pressure guidewire was calibrated and pushed to the tip of the guide catheter to equalize the pressure and temperature signals. The pressure guidewire was sent to the distal target lesion until the pressure sensor was placed about 3 cm away from the distal lesion. Then, 200 μg nitroglycerin was injected into the coronary artery and measurement was performed after the pressure curve was stable. The mean distal coronary artery pressure (Pd at wave-free period) and mean aortic pressure (Pa at wave-free period) during the diastolic wave-free period (25% after the beginning of diastolic period to 5 ms before the end of diastolic period) were obtained from the coronary functional assessment system, and iFR was calculated as the mean Pd divided by the mean Pa during the diastolic wave-free period. In addition, FFR was calculated by dividing the coronary pressure measured with the sensor placed distal to the stenosis (Pd) by the aortic pressure measured through the guide catheter (Pa) under maximum hyperemic conditions, which could be conducted by intravenous injection of adenosine (140 μg/kg/min). An iFR value ≤ 0.89 or a FFR value ≤ 0.80 was hemodynamically considered significant. Finally, CCTA, ICA, iFR, and FFR data were transferred to the central core laboratory for further analysis.

### Computation of AccuiFRct

The AccuiFRct was calculated in a blinded manner using a workstation-based software (ArteryFlow Technology Co., Ltd.).

The centerlines of coronary arteries were obtained from CCTA data. A 3D dilated convolutional neural network (CNN) was previously trained using a training set consisting of 100 CCTA images with 500 centerlines to predict coronary artery centerlines in CCTA using manually annotated centerlines as the reference standard. In the validation group of 50 CCTA scans with 250 centerlines, the extracted centerlines had an average overlap of 94% with manually annotated reference centerlines and the extracted centerline points were highly accurate, with an average distance of 0.2 mm to reference centerline points. With the CNN extracted centerlines of the coronary artery tree, the 3D model of coronary arteries was reconstructed by coupling the optimal vessel borders and the centerline in space coordinates. On the basis of the 3D model, a mesh model was further established for simulation of CFD, in which a finite volume approach was applied to solve the Navier–Stokes equations. Resting blood flow at the inlet was measured by the relationship between the left ventricle myocardial mass and blood flow rate. As for outlet boundaries, the Murray’s law was employed to calculate flow rate in each branch by lumen diameters, and the corresponding blood flow rates were then set as the outlet boundary conditions. Blood was modelled as an incompressible Newtonian fluid with a density of 1056 kg/m^3^ and a viscosity of 0.0035 Pa s in the simulation of CFD.

The simulation was steady and the vessel wall was assumed to be rigid with a no-slip boundary condition. The numerical results of the measurement of pressure and velocity were computed and visualized on the 3D coronary artery tree. The AccuiFRct was computed as the mean pressure distal to the stenosis during the diastolic wave-free period divided by the mean aortic pressure during the diastolic wave-free period. Patient-specific Pa was derived from patient-individualized pressure data, in which Pa was equal to the diastolic blood pressure at the aortic root. Thus, the distribution of AccuiFRct at any position of the 3D coronary artery tree could be obtained. The total processing time, including reconstruction and CFD simulation, was around 30 min.

### Statistical analysis

Statistical analysis was performed using the SPSS 20.0 (IBM Corp., Armonk, NY, USA), MedCalc 19.0.4 (MedCalc Software Inc., Ostend, Belgium) software and R software, version 4.1.1 (R Project for Statistical Computing). Continuous variables were presented as mean ± standard deviation. Abnormally distributed data were reported as median (interquartile range [IQR]). A linear regression model was used to analyze the correlation between indices. The differences in correlation coefficients were compared by the Fisher’s exact test. The agreement between AccuiFRct and FFR or between AccuiFRct and iFR was tested by Bland–Altman plots. Diagnostic performance of AccuiFRct was presented with sensitivity, specificity, positive predictive value (PPV), negative predictive value (NPV), and diagnostic accuracy. Discriminant function was assessed using area under the curve (AUC) and 95% confidence intervals (CIs) in receiver operating characteristic (ROC) curve analysis, and the values of AUC were compared using the DeLong method. A decision curve analysis (DCA) was used to demonstrate the net benefit of AccuiFRct. All probability values were 2-sided, and *P* < 0.05 was considered statistically significant.

## Results

### Patients’ demographic and clinical characteristics at baseline

**Fig. 1:**
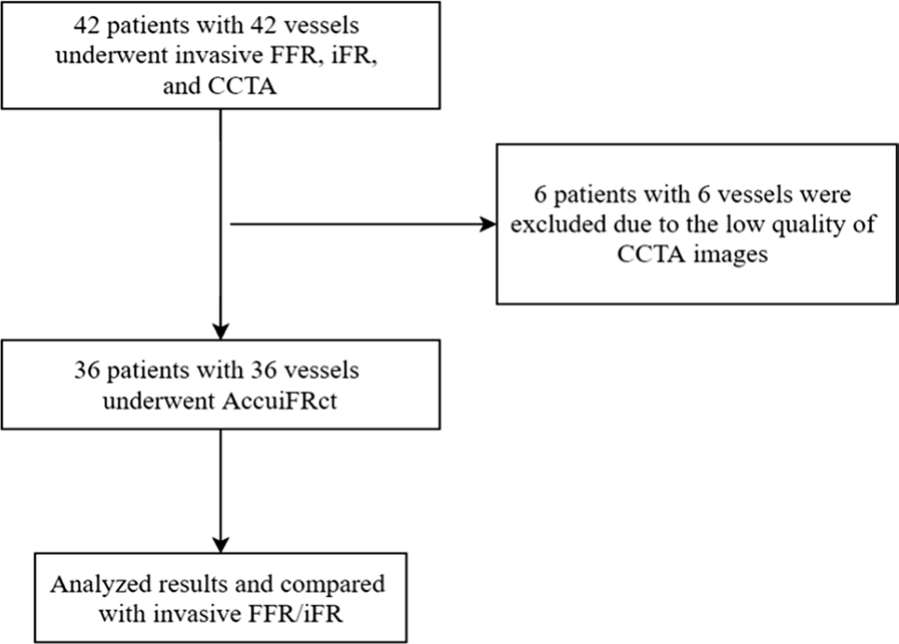
Participant flow chart of the study. FFR, fractional flow reserve; AccuiFRct, instantaneous wave-free ratio derived from computed tomography; CCTA, coronary computed tomographic angiography

In this study, we included 42 patients (with 42 vessels) who underwent CCTA, ICA, iFR, and FFR measurement, in which 6 vessels were excluded for AccuiFRct computation due to poor CCTA image quality: unsatisfied resolution leading to unsuccessful reconstruction of the investigated vessel (n = 3), failed reconstruction due to the presence of severe motion artifact (n = 1) and image discontinuity (n = 2). The final study population consisted of 36 patients with 36 vessels (Fig. 1). Patients’ demographic and clinical characteristics at baseline are shown in Table 1. Patients’ median age was 67.8 ± 7.9 years old, and 28 (78%) were men. The vessels included 26 left anterior descending coronary arteries (LAD, 72%), 4 left circumflex coronary arteries (LCX, 11%), and 6 right coronary arteries (RCA, 17%). The mean CCTA–derived of the lesions was 44.3 ± 12.3%. The mean values of FFR and iFR were 0.80 ± 0.10 and 0.91 ± 0.06, respectively. The proportions of FFR ≤ 0.80 and iFR ≤ 0.89 were 42% and 39%, respectively.

**Table 1:** Baseline patient and lesion characteristics

Parameter	Number of patients (36)
Age (years)	67.8 ± 7.9
Men, n (%)	28 (78)
Weight (kg)	68.8 ± 10.3
Height (cm)	166.3 ± 7.2
Body mass index (kg/m^2^)	24.8 ± 3.2
*Cardiovascular risk factors, n(%)*	
Hypertension	27 (75)
Diabetes mellitus	11 (31)
Hypercholesterolemia	8 (22)
Current smoker	16 (44)
LVEF (%)	65.53 ± 6.46
EDV (mL)	110.65 ± 22.63
ESV (mL)	38.31 ± 11.31
LVIDd (mm)	4.83 ± 0.43
LVIDs (mm)	3.08 ± 0.37
Lesion location, n (%)	
LAD	26 (72)
LCX	4 (11)
RCA	6 (17)
Coronary CT angiography	
Agatston score, n (%)	
0–399	29 (81)
400–799	1 (3)
> 799	6 (17)
CCTA stenosis ≥ 50%	10 (28)
Invasive physiologic indices	
FFR	0.80 ± 0.10
iFR	0.91 ± 0.06
Noninvasive physiologic indices	
AccuiFRct	0.90 (0.97–0.95)
AccuiFRct≤0.89	15 (42)
iFR ≤ 0.89	14 (39)
FFR ≤ 0.80	15 (42)

*LVEF*, left ventricular ejection fraction; *EDV*, end diastolic volume; *LVIDd*, left ventricular internal diameter at end-diastole; *LVIDs*, left ventricular internal diameter at end-systole; *ESV*, end systolic volume; *LAD*, left anterior descending artery; *LCX*, left circumflex artery; *RCA*, right coronary artery; *CT*, computed tomography; *CCTA*, coronary computed tomography angiography; *FFR*, fractional flow reserve; *iFR*, instantaneous wave-free ratio; *AccuiFRct*,instantaneous wave-free ratio derived from coronary computed tomography angiography

### Correlation and agreement between AccuiFRct and FFR, AccuiFRct and iFR

**Fig. 2:**
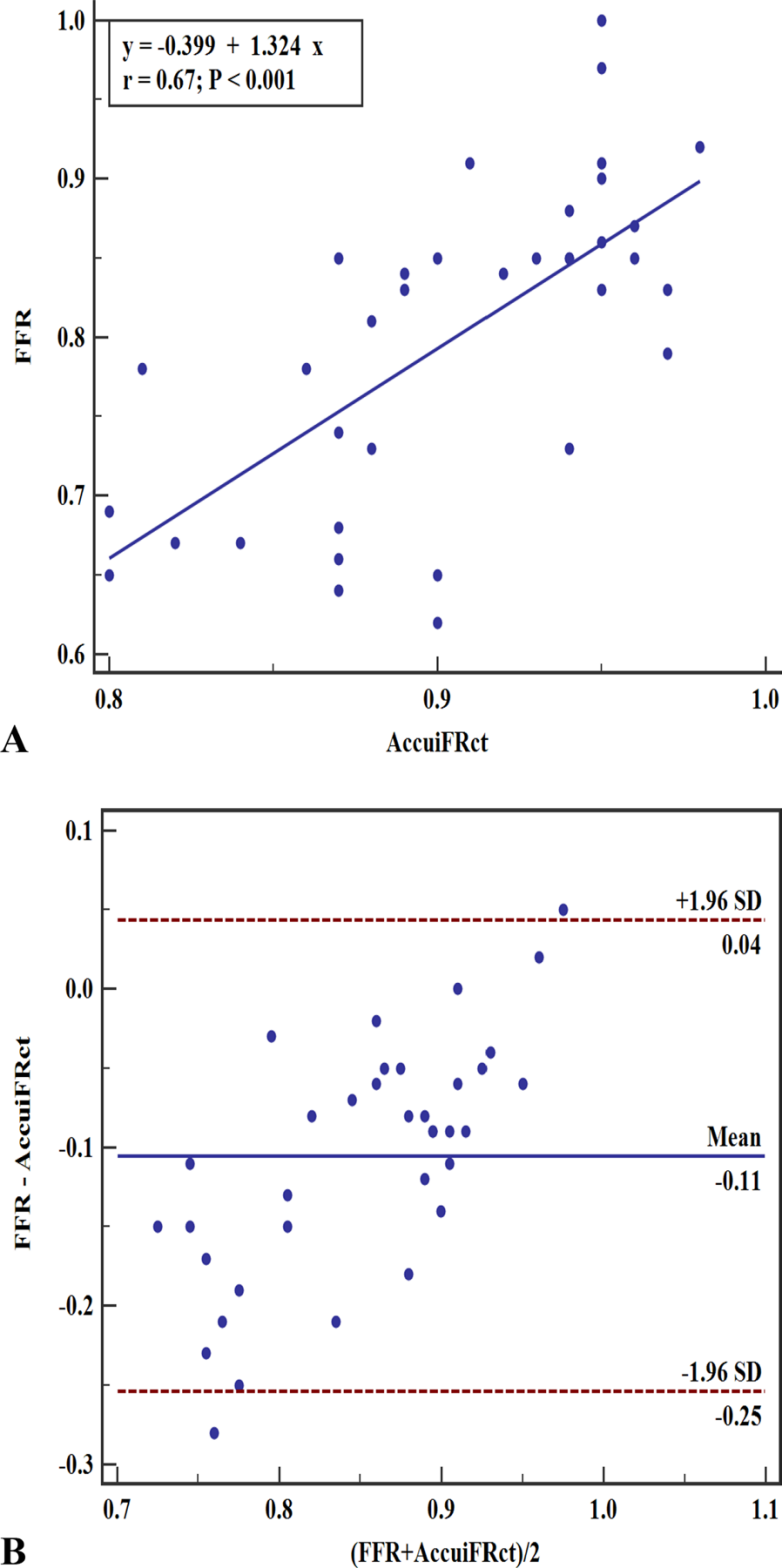
Correlation and agreement between AccuiFRct and FFR. **A** High correlation between AccuiFRct and FFR. **B** Good agreement between AccuiFRct and FFR. Mean value of AccuiFRct minus FFR = 0.106; upper limit of agreement =  − 0.254; lower limit of agreement = 0.043. FFR, fractional flow reserve; AccuiFRct, instantaneous wave-free ratio derived from computed tomography

**Fig. 3:**
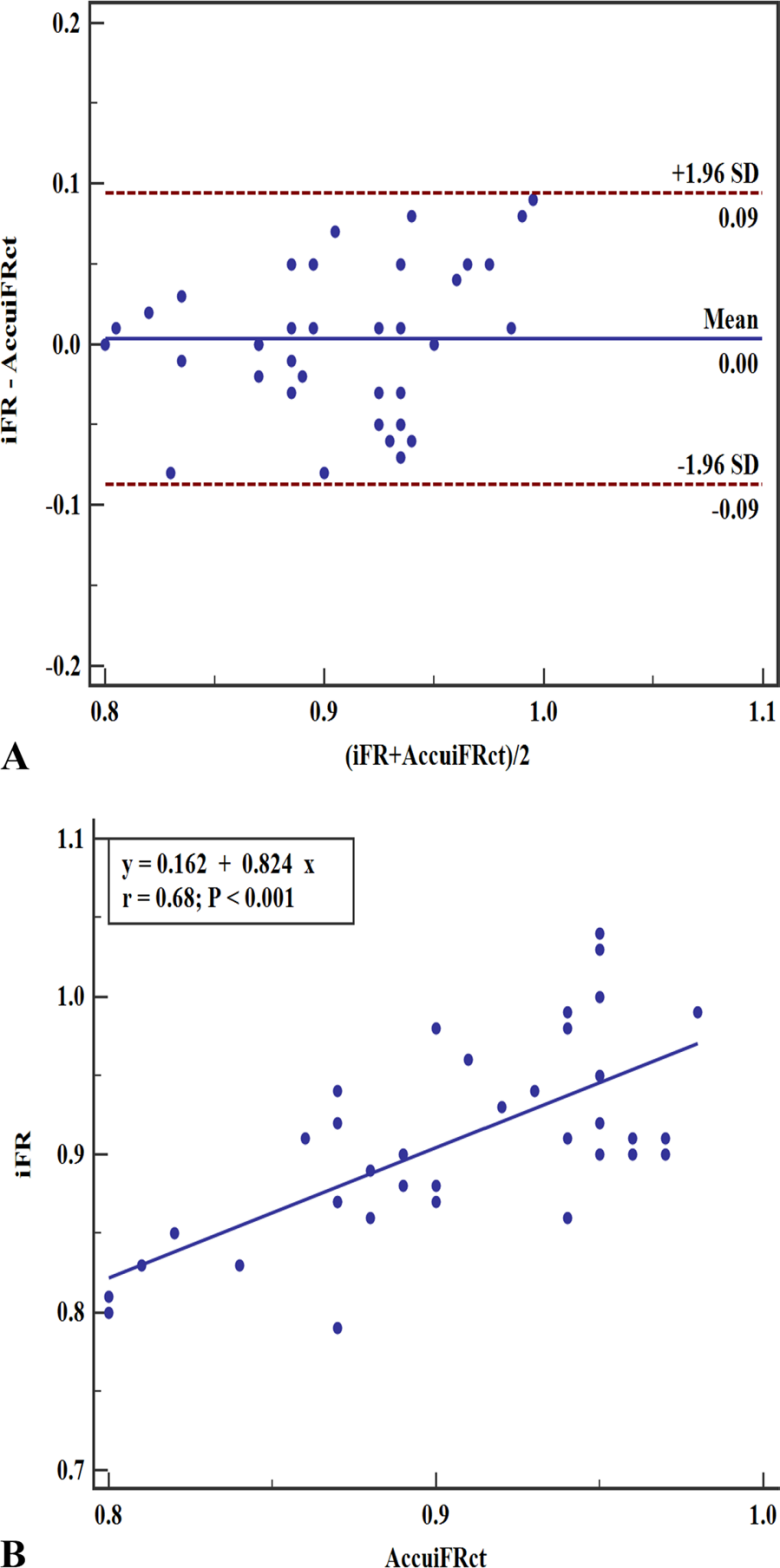
Correlation and agreement between AccuiFRct and iFR. **A** Good agreement between AccuiFRct and iFR. Mean value of AccuiFRct minus iFR = − 0.003; upper limit of agreement = 0.094; lower limit of agreement = − 0.087. **B** High correlation between AccuiFRct and iFR. iFR, instantaneous wave-free ratio; AccuiFRct, instantaneous wave-free ratio derived from computed tomography

**Fig. 4:**
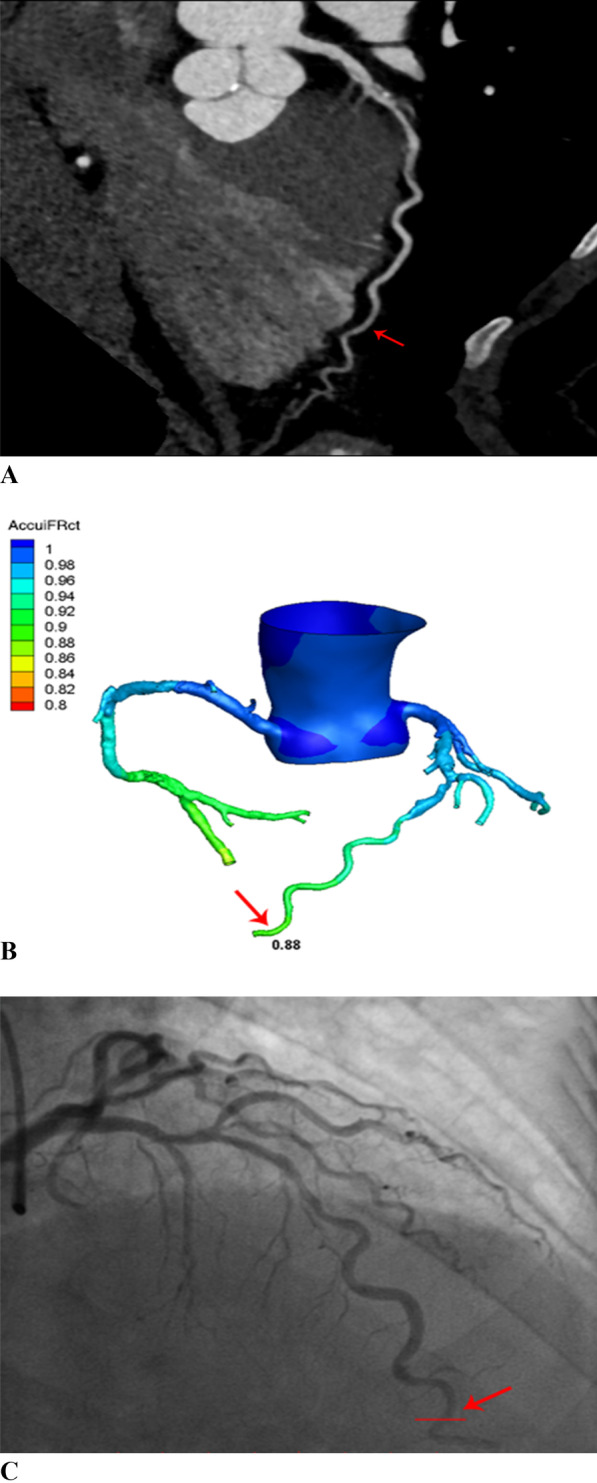
**A** CCTA demonstrating 41% stenosis at the portion of LAD (red arrow); **B** a computed AccuiFRct value of 0.88 (red arrow); **C** Coronary catheter angiography shows a stenosis (red arrow) with an iFR measurement of 0.86. iFR, instantaneous wave-free ratio; AccuiFRct, instantaneous wave-free ratio derived from computed tomography; CCTA coronary computed tomography angiography, LAD left anterior descending artery

In the majority of blood vessels, the AccuiFRct value was higher than the FFR value. Besides, AccuiFRct had a moderately linear correlation with FFR and iFR, presenting a similar correlation coefficient (r = 0.67 versus 0.68, *P* = 0.941) (Fig. 2 and Fig. 3). Representative example of anatomically obstructive stenosis without ischemia-producing stenosis is shown in Fig. 4. The agreement between AccuiFRct and iFR was better than that between AccuiFRct and FFR in the total population (bias ± SD: 0.003 ± 0.046 vs. 0.106 ± 0.076, *P* < 0.001) (Figs. 2, 3). The mean time for AccuiFRct assessment (including 3D reconstruction based on CCTA images and frame count analysis) was about 30 min.

### Diagnostic performance of AccuiFRct for predicting FFR or iFR

**Fig. 5:**
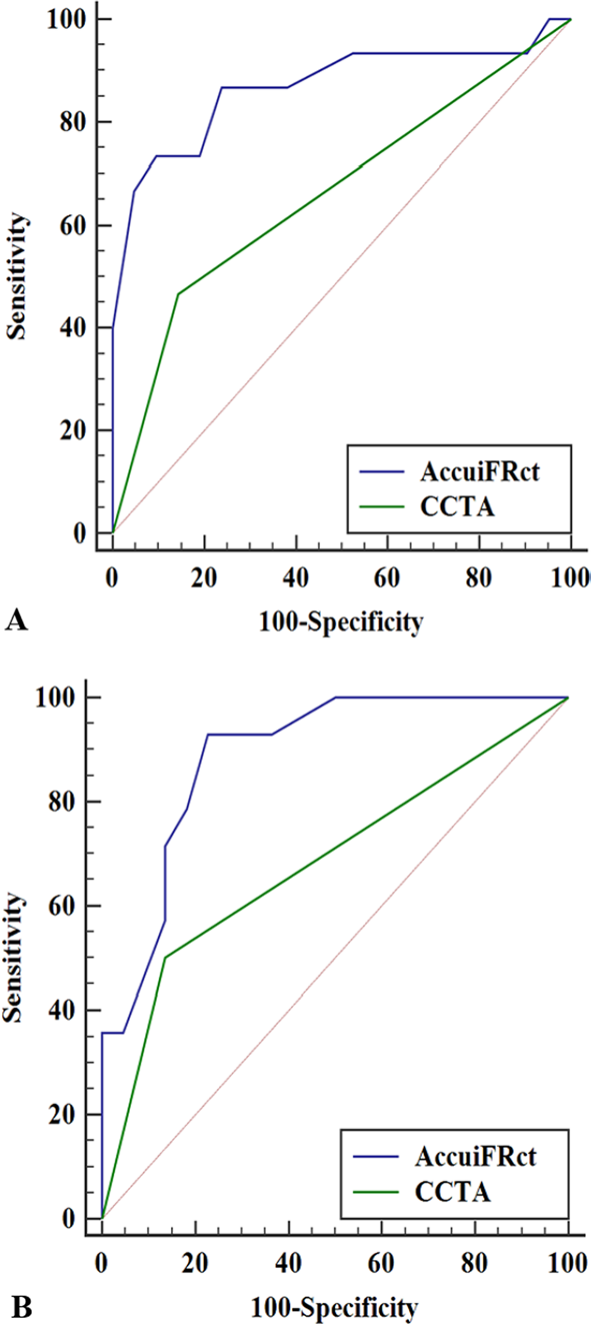
Overall diagnostic accuracy (area under the curve in receiver operating characteristic analysis) of AccuiFRct in detecting FFR ≤ 0.80 (**A**) and iFR ≤ 0.89 (**B**). iFR, instantaneous wave-free ratio; FFR, fractional flow reserve; AccuiFRct, instantaneous wave-free ratio derived from computed tomography

The discriminant function of AccuiFRct to predict FFR ≤ 0.80 or iFR ≤ 0.89 is illustrated in Fig. 5. The AUC values of diameter stenosis (DS% ≥ 50%) and AccuiFRct for FFR ≤ 0.80 were 0.66 (95% CI 0.49–0.81) and 0.87 (95% CI 0.71–0.96), respectively (*P* < 0.001). The AUC values of DS% ≥ 50% and AccuiFRct for iFR ≤ 0.89 were 0.68 (95% CI 0.51–0.83) and 0.89(95% CI 0.74–0.97), respectively (*P* < 0.001). Parameters describing diagnostic performance of AccuiFRct to predict FFR ≤ 0.80 or iFR ≤ 0.89 are summarized in Table 2. Using FFR ≤ 0.8 as a reference, the sensitivity, specificity, PPV, NPV, and diagnostic accuracy of AccuiFRct were 73%, 81%, 73%, 81%, and 78%, respectively. With iFR as a reference, the sensitivity, specificity, PPV, NPV, and diagnostic accuracy of AccuiFRct were 73%, 86%, 79%, 82%, and 81%, respectively. Numerically, the values of all diagnostic indices of AccuiFRct were higher when iFR was used as a reference rather than FFR, except for the same sensitivity.

**Table 2:** Comparison of diagnostic performance of AccuiFRct to Predict FFR or iFR

	FFR as reference	iFR as reference
True positive	11	11
True negative	17	18
False positive	4	3
False negative	4	4
Sensitivity, %	73% (44.83–91.09)	73% (44.83–91.09)
Specificity, %	81% (57.42–93.71)	86% (62.64–96.24)
PPV, %	73% (44.83–91.09)	79% (48.82–94.29)
NPV, %	81% (57.42–93.71)	82% (58.99–94.01)
Diagnostic accuracy, %	78% (47.62–89.54)	81% (51.68–93.16)
AUC	0.87 (0.71–0.96)	0.89 (0.74–0.97)

Values are expressed as estimates with 95% CIs. Sensitivity, specificity, positive predictive value (PPV), negative predictive value (NPV), and diagnostic accuracy were calculated based on per-vessel analysis. FFR, fractional flow reserve; iFR, instantaneous wave-free ratio; AccuiFRct, instantaneous wave-free ratio derived from computed tomography

### Decision curve analysis for AccuiFRct

Decision curve analysis (DCA) is a method for evaluating the benefit of diagnostic tests, prediction models or molecular markers. Traditional statistical measures such as sensitivity, specificity, and area under the ROC curve only evaluate the diagnostic performance of the model but fail to provide an answer as to the clinical utility of a specific model, the advantage of DCA is that it integrates the likely range of a patient’s risk and benefit preferences into the consideration. DCA calculates a clinical “net benefit” for one or more prediction models or diagnostic tests in comparison to default strategies of treating all or no patients. Net benefit = true positive rate − (false positive rate × weighting factor), where weighting factor = threshold probability/(1 − threshold probability) [20].

**Fig. 6:**
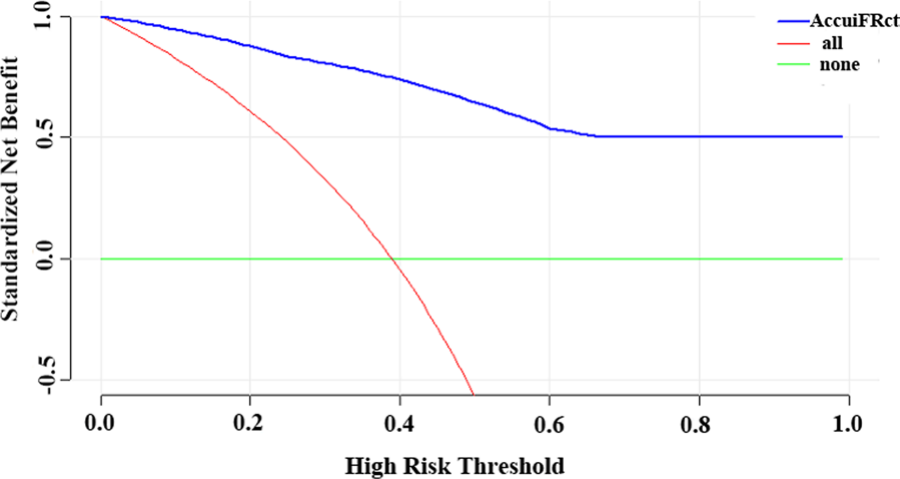
Decision curve analysis for AccuiFRct predicting myocardial ischemia. The red line indicates myocardial ischemia in all patients. The green line is the absence of myocardial ischemia in all patients. The decision curve shows that: AccuiFRct has a higher net benefit compared to the clinical strategies of “all” or “none”, over the entire range of reasonable threshold probabilities. Using AccuiFRct to decide myocardial ischemia would therefore lead to the better clinical outcomes

The decision curves for default strategies and for AccuiFRct were shown in Fig. 6. In the DCA, AccuiFRct had a much higher net benefit than default strategies which was over 50% through the entire range of reasonable threshold probabilities, indicating that the use of AccuiFRct can leads to better decisions.

## Discussion

We developed a novel method that allows fast computation of iFR from conventional CCTA images alone. The present study assessed the diagnostic performance of AccuiFRct to define physiologically significant coronary stenosis using FFR or iFR as reference standard methods. The main findings of our study are summarized in the following: (a) the AccuiFRct showed a good correlation and a promising diagnostic performance for both invasive pressure-derived physiological indices (FFR and iFR); (b) when iFR was used as the reference standard, the diagnostic accuracy and discriminant function of AccuiFRct were numerically higher; (c) AccuiFRct had a better diagnostic performance for hemodynamic significance of coronary stenosis compared to CCTA alone; (d) owing to the high degree of automation using artificial intelligence, AccuiFRct analysis was conducted in a short computational time. AccuiFRct considers both anatomical and functional data, enabling a comprehensive assessment of the influences of lesions on blood flow, as well as facilitating in making reliable clinical decisions.

To date, few studies have explored the feasibility of CCTA-derived iFR by CFD method based on vessel volume of coronary arteries and Murray’s law for improving the diagnostic efficiency for the physiological assessment of coronary lesions [18, 19]. In 2017, Ma et al. proposed a numerical method to calculate iFR based on conventional CCTA images, and they achieved overall accuracy, sensitivity, and specificity of 79%, 71%, and 83%, respectively [18]. In 2018, Ma et al. used the same computational method to perform another study involving 39 patients (55 vessels), and the diagnostic accuracy, sensitivity, and specificity were 75%, 85%, and 69%, respectively [19]. However, an anatomical model of the coronary artery was established using Mimics 10.1 software (Materialise, Leuven, Belgium). In addition, due to technical limitations, the diagnostic performance of iFR_CT_ in predicting FFR ≤ 0.80 was evaluated, exclusively. Moreover, the iFR_CT_ optimal threshold values for the diagnosis of functional stenosis were different in their study (optimal cutoff values were 0.82 and 0.85, respectively).

Our study optimized a method for fast computation of iFR using the conventional CCTA images without requiring a pressure guidewire and hyperemic agents. A good correlation between AccuiFRct and invasive FFR was observed (r = 0.67, *P* < 0.001), which was similar to r = 0.65 obtained by Ma et al. The AUC value was higher for AccuiFRct than that for DS% (0.87 vs. 0.67). The overall diagnostic accuracy of AccuiFRct (78%) to predict FFR in our study was comparable to that reported previously [18, 19]. The results of the present study revealed the high efficiency of using AccuiFRct for the diagnosis of coronary artery stenosis, supporting the use of iFR as a pressure index for identifying hemodynamic significance of coronary stenosis.

Our results confirmed the high efficiency of using AccuiFRct for the diagnosis of functional stenosis. The diagnostic accuracy, sensitivity, specificity, PPV, and NPV of AccuiFRct ≤ 0.89 for predicting FFR were 78%, 73%, 81%, 73%, and 81%, respectively. Such results are mostly comparable to the diagnostic performance of FFR_CT_ reported in previous prospective studies. Other multicenter researches have demonstrated that FFR_CT_ was strongly correlated with FFR (r = 0.72–0.82, *P* < 0.001). The diagnostic accuracy, sensitivity, and specificity were 81–87%, 86–92%, and 79–81%, respectively [12–14]. The present study confirmed the feasibility of AccuiFRct in a clinical setting, thereby providing a novel noninvasive index. Additionally, the new method does not need to simulate the steps of maximum hyperemia, and it is closer to the true physiological state during CCTA scanning.

To our knowledge, iFR is a pressure ratio measured during the wave-free period without hyperemia [5]. A number of scholars pointed out that certain hemodynamics and lesion characteristics could influence discordance between iFR and FFR [21]. The incidence of disagreement between FFR and iFR was reported to be 10–30% [22]. VERIFY study indicated a weak correlation between iFR and FFR, and iFR cannot be therefore recommended for making reliable clinical decisions in patients with CAD [23]. However, two multicenter trials found that iFR-guided revascularization was comparable to FFR-guided revascularization [7, 8]. Another study highlighted the complementariness between iFR and FFR and supported the iFR-FFR hybrid strategy, avoiding adenosine administration in 69% of stenoses without compromising diagnostic accuracy. This prospective multicenter study confirmed the feasibility of iFR in clinical practice [24]. Previous multicenter studies have demonstrated diagnostic accuracy, sensitivity, specificity, PPV, and NPV of iFR equal to 80–88%, 73–85%, 82–91%, 77–91%, and 73–85%, respectively [5, 24, 25]. The PACIFIC trial proved that FFR and iFR were similar to [^15^O] H_2_O positron emission tomography (PET) perfusion imaging in assessing functional significant lesions [26]. In addition, CLARIFY study demonstrated that iFR and FFR have an equal diagnostic classification agreement with hyperemic stenosis resistance [27]. Choi et al. showed the feasibility of iFR-guided treatment for no-culprit stenoses in the acute stage of acute myocardial infarction (AMI) [28]. Moreover, Rosa et al. proved that the measurement of iFR is reliable in patients with left main coronary artery disease [29]. This adenosine-independent approach possesses several advantages over the invasive FFR. Consequently, iFR was also included in the recent guidelines. iFR_CT_ has not been comprehensively evaluated using other reference standards other than FFR. As the existing guidelines have recommended the use of iFR to guide decisions as class IA recommendations, it is important to use iFR as a reference standard to assess the diagnostic ability of AccuiFRct in defining significant functional stenosis.

During the computation of CT-derived FFR, boundary conditions would be converted from baseline to hyperemic to simulate the drug-induced hyperemia and to match the clinical meaning of FFR. On the contrary, AccuiFRct could simulate the coronary flow under the same clinical conditions, and no conversion error would be introduced, which might be an alternative option for hemodynamic assessment of coronary stenosis. In contrast to the calculation of FFR_CT_ and measurement of FFR, neither the AccuiFRct nor iFR need to simulate maximum blood filling, which is closer to the true physiological state to mimic the clinical settings when the diagnostic performance of iFR_CT_ and iFR is compared. In the present study, we examined an AccuiFRct CFD algorithm in terms of clinical feasibility for the determination of the functional significance of coronary lesions compared to the current invasive reference standard of iFR. To the best of our knowledge, this study is the first to compare AccuiFRct based on CCTA images by a deep-learning-based approach with iFR. Our results showed that the AccuiFRct algorithm outperformed in clinical settings. In addition, on average, only 30 min was required for the whole workflow, including 3D reconstruction of coronary artery geometry and CFD simulation. This is mainly due to the CNN-based deep-learning reconstruction model, in which the 3D anatomical coronary artery model could be established within 5 min without any complex interaction. Time efficiency is a key factor in diagnosing patients with CAD, and shortening diagnosis time is of great clinically significance. The optimal cut-off values for OCT-FFR and IVUS-FFR were 0.80 [30, 31]. As a result, we used AccuiFRct ≤ 0.89 as the optimal threshold for predicting ischemic lesions. Similar to a previous study, we found a substantial increase in the AUC value on a per-lesion level [AUC, 0.89 (95% CI: 0.74–0.97)] to detect hemodynamically significant coronary stenosis compared to the evaluation of CCTA studies alone. DCA also indicated that AccuiFRct had clinical value in predicting significance stenosis. In the current study, with the use of iFR as the reference standard, AccuiFRct showed a more promising diagnostic performance and a discriminant function for detecting hemodynamically relevant stenosis. Furthermore, our results revealed a good correlation between AccuiFRct and iFR. Thus, the results of this study support the clinical value of AccuiFRct in assessing the functional significance of coronary stenoses from the iFR point of view.

A previous study evaluated an on-site machine learning-based FFR_CT_ approach in terms of diagnostic performance compared to invasive iFR (correlation coefficient, 0.82; accuracy, 93%; sensitivity, 87%; and specificity, 95%) [32]. However, because of the small sample size, our study could not compare the diagnostic performance of AccuiFRct, FFR_CT_ and iFR.

### Clinical implications

We, for the first time, reported a correlation between AccuiFRct and iFR. It was revealed that AccuiFRct is more appropriate for comparing with iFR. AccuiFRct does not need to simulate the maximum blood filling, while FFR is calculated under the maximum hyperemia state, thus, the blood flow conditions calculated by the two are inconsistent. The comparison between AccuiFRct and iFR, on the one hand, can more accurately explain the diagnostic efficiency of AccuiFRct; on the other hand, the diagnostic accuracy of AccuiFRct can be explained from both iFR and FFR perspectives.

In our study, 3D coronary artery tree and boundary conditions are based on deep learning method, greatly reducing the calculation time. AccuiFRct possesses several advantages. AccuiFRct was obtained from CCTA data during the diastolic wave-free period of the resting state, indicating that hemodynamic significance of coronary lesions could be identified without drug-induced hyperemia. This could be advantageous, particularly for patients who have adenosine intolerance. Compared with a similar approach for assessing ischemia from CCTA, FFR_CT_, there is no need for converting the resting data to hyperemic in AccuiFRct because the conditions of CFD simulation and the realistic patient-specific state were the same. On the other hand, errors could be induced during the conversion in FFR_CT_-dependent approaches. In the diagnosis of myocardial ischemia, AccuiFRct has a good diagnostic performance. The calculation workflow of AccuiFRct is time-saving and convenient. The clinical roles of AccuiFRct are different from FFR, iFR, and QFR. In contrast, AccuiFRct can be primarily used in outpatient departments, reducing the number of unnecessary coronary angiographic studies in patients without functionally significant ischemic lesions.

### Study limitations

First, this was a retrospective, single-center study, hindering the generalization of the results. Second, 6 patients were excluded due to the poor quality of CCTA images. However, the successful AccuiFRct computation was acquired in 36 of 42 (86%) lesions in our study, which is in line with previous studies showing that there were about 10–15% of patient cases can not be used because of the poor quality of CCTA images [13, 14]. Third, the sample size of this study was small and this study included only patients with stable CAD. There are about 1600 patients with stable CAD admitted to our hospital during the 2-year study period. It is mainly due to the low adoption rate of FFR or iFR caused by the cost of intracoronary pressure guidewires, prolonged procedural time and adenosine-induced adverse reactions. The adoption rates of FFR or iFR is less than 6% in many countries including China [11], and the adoption rate of FFR or iFR is about 8% of the clinical practice in our catheter lab during the study period. The number of patients who had undergone CCTA and invasive physiological measurements both FFR and iFR within 2 months is 46. Therefore, more prospective large-sample trials are needed to adequately verify the diagnostic performance of AccuiFRct. Fourth, follow-up data were not evaluated, therefore, we could not assess the prognostic implications of AccuiFRct-guided treatment. In the future research, AccuiFRct should be further studied with MACE events as the primary endpoint. Fifth, the effects of calcified plaques and coronary artery morphology on the computational accuracy of AccuiFRct were not herein clarified, while Ma et al. showed that iFR_CT_ had a good diagnostic performance for functional stenosis caused by markedly calcified plaques [18].

## Conclusions

In the present study, a novel CCTA-derived iFR method, AccuiFRct, showed a promising diagnostic accuracy compared with iFR as a resting index; moreover, a good correlation between AccuiFRct and iFR in the detection hemodynamically significant stenosis could be observed. However, further multicenter prospective studies are necessary to eliminate the above-mentioned deficiencies and to validate our findings.

## Data Availability

The data that support the findings of this study are available from the corresponding author upon reasonable request.

## Abbreviations

FFR: Fractional flow reserve
iFR: Instantaneous wave-free ratio
CFD: Computational fluid dynamics
CCTA: Coronary computed tomography angiography
ICA: Invasive coronary angiography
MACE: Major adverse cardiac events
CAD: Coronary artery disease
LVEF: Left ventricular ejection fraction
CNN: Convolutional neural network
PPV: Positive predictive value
NPV: Negative predictive value
AUC: Area under the curve
ROC: Receiver operating characteristic
LAD: Left anterior descending artery
LCX: Left circumflex coronary arteries
RCA: Right coronary arteries
PET: Positron emission tomography
AMI: Acute myocardial infarction
DCA: Decision curve analysis

## References

[1] Knuuti J, Wijns W, Saraste A (2020). 2019 ESC Guidelines for the diagnosis and management of chronic coronary syndromes. Eur Heart J.

[2] Kofoed KF, Engstrøm T, Sigvardsen PE (2021). Prognostic value of coronary CT angiography in patients with non-ST-segment elevation acute coronary syndromes. J Am Coll Cardiol.

[3] Kogame N, Ono M, Kawashima H (2020). The impact of coronary physiology on contemporary clinical decision making. JACC Cardiovasc Interv.

[4] Windecker S, Kolh P, Alfonso F (2014). 2014 ESC/EACTS guidelines on myocardial revascularization: the Task Force on Myocardial Revascularization of the European Society of Cardiology (ESC) and the European Association for Cardio-Thoracic Surgery (EACTS) Developed with the special contribution of the European Association of Percutaneous Cardiovascular Interventions (EAPCI). Eur Heart J.

[5] Sen S, Escaned J, Malik IS (2012). Development and validation of a new adenosine-independent index of stenosis severity from coronary wave-intensity analysis: results of the ADVISE (ADenosine Vasodilator Independent Stenosis Evaluation) study. J Am Coll Cardiol.

[6] Petraco R, van de Hoef TP, Nijjer S (2014). Baseline instantaneous wave-free ratio as a pressure-only estimation of underlying coronary flow reserve: results of the JUSTIFY-CFR Study (Joined Coronary Pressure and Flow Analysis to Determine Diagnostic Characteristics of Basal and Hyperemic Indices of Functional Lesion Severity-Coronary Flow Reserve). Circ Cardiovasc Interv.

[7] Davies JE, Sen S, Dehbi HM (2017). Use of the instantaneous wave-free ratio or fractional flow reserve in PCI. N Engl J Med.

[8] Götberg M, Christiansen EH, Gudmundsdottir IJ (2017). Instantaneous wave-free ratio versus fractional flow reserve to guide PCI. N Engl J Med.

[9] Neumann FJ, Sousa-Uva M, Ahlsson A (2019). 2018 ESC/EACTS guidelines on myocardial revascularization. Eur Heart J.

[10] Levine GN, Bates ER, Blankenship JC (2011). 2011 ACCF/AHA/SCAI guideline for percutaneous coronary intervention. A report of the American College of Cardiology Foundation/American Heart Association Task Force on Practice Guidelines and the Society for Cardiovascular Angiography and Interventions. J Am Coll Cardiol.

[11] Götberg M, Cook CM, Sen S (2017). The evolving future of instantaneous wave-free ratio and fractional flow reserve. J Am Coll Cardiol.

[12] Koo BK, Erglis A, Doh JH (2011). Diagnosis of ischemia-causing coronary stenoses by noninvasive fractional flow reserve computed from coronary computed tomographic angiograms. Results from the prospective multicenter DISCOVER-FLOW (Diagnosis of Ischemia-Causing Stenoses Obtained Via Noninvasive Fractional Flow Reserve) study. J Am Coll Cardiol.

[13] Nørgaard BL, Leipsic J, Gaur S (2014). Diagnostic performance of noninvasive fractional flow reserve derived from coronary computed tomography angiography in suspected coronary artery disease: the NXT trial (Analysis of Coronary Blood Flow Using CT Angiography: Next Steps). J Am Coll Cardiol.

[14] Min JK, Leipsic J, Pencina MJ (2012). Diagnostic accuracy of fractional flow reserve from anatomic CT angiography. JAMA.

[15] Celeng C, Leiner T, Maurovich-Horvat P (2019). Anatomical and functional computed tomography for diagnosing hemodynamically significant coronary artery disease: a meta-analysis. JACC Cardiovasc Imaging.

[16] Fairbairn TA, Nieman K, Akasaka T (2018). Real-world clinical utility and impact on clinical decision-making of coronary computed tomography angiography-derived fractional flow reserve: lessons from the ADVANCE Registry. Eur Heart J.

[17] Patel MR, Nørgaard BL, Fairbairn TA (2020). 1-Year impact on medical practice and clinical outcomes of FFRCT: the ADVANCE registry. JACC Cardiovasc Imaging.

[18] Ma Y, Liu H, Hou Y (2017). Instantaneous wave-free ratio derived from coronary computed tomography angiography in evaluation of ischemia-causing coronary stenosis: feasibility and initial clinical research. Medicine.

[19] Ma Y, Hou Y, Qiao A, Jing Q (2018). Non-invasive instantaneous wave-free ratio using coronary CT angiography: diagnostic performance for evaluation of ischaemia-causing coronary stenosis confirmed by invasive fractional flow reserve. Clin Radiol.

[20] Fitzgerald M, Saville BR, Lewis RJ (2015). Decision curve analysis. JAMA.

[21] Arashi H, Satomi N, Ishida I (2019). Hemodynamic and lesion characteristics associated with discordance between the instantaneous wave-free ratio and fractional flow reserve. J Interv Cardiol.

[22] Lee JM, Doh JH, Nam CW (2018). Functional approach for coronary artery disease: filling the gap between evidence and practice. Korean Circ J.

[23] Berry C, van ’t Veer M, Witt N (2013). VERIFY (VERification of Instantaneous Wave-Free Ratio and Fractional Flow Reserve for the Assessment of Coronary Artery Stenosis Severity in EverydaY Practice): a multicenter study in consecutive patients. J Am Coll Cardiol.

[24] Escaned J, Echavarría-Pinto M, Garcia-Garcia HM (2015). Prospective assessment of the diagnostic accuracy of instantaneous wave-free ratio to assess coronary stenosis relevance: results of ADVISE II International, Multicenter Study (ADenosine Vasodilator Independent Stenosis Evaluation II). JACC Cardiovasc Interv.

[25] Jeremias A, Maehara A, Généreux P (2014). Multicenter core laboratory comparison of the instantaneous wave-free ratio and resting Pd/Pa with fractional flow reserve: the RESOLVE study. J Am Coll Cardiol.

[26] de Waard GA, Danad I, Petraco R (2018). Fractional flow reserve, instantaneous wave-free ratio, and resting Pd/Pa compared with [^15^O]H_2_O positron emission tomography myocardial perfusion imaging: a PACIFIC trial sub-study. Eur Heart J.

[27] Sen S, Asrress KN, Nijjer S (2013). Diagnostic classification of the instantaneous wave-free ratio is equivalent to fractional flow reserve and is not improved with adenosine administration. Results of CLARIFY (Classification Accuracy of Pressure-Only Ratios Against Indices Using Flow Study). J Am Coll Cardiol.

[28] Choi KH, Lee JM, Kim HK (2018). Fractional flow reserve and instantaneous wave-free ratio for nonculprit stenosis in patients with acute myocardial infarction. JACC Cardiovasc Interv.

[29] De Rosa S, Polimeni A, De Velli G (2019). Reliability of instantaneous wave-free ratio (iFR) for the evaluation of left main coronary artery lesions. J Clin Med.

[30] Lee KE, Lee SH, Shin ES, Shim EB (2017). A vessel length-based method to compute coronary fractional flow reserve from optical coherence tomography images. Biomed Eng Online.

[31] Yu W, Tanigaki T, Ding D (2021). Accuracy of intravascular ultrasound-based fractional flow reserve in identifying hemodynamic significance of coronary stenosis. Circ Cardiovasc Interv.

[32] Baumann S, Hirt M, Schoepf UJ (2020). Correlation of machine learning computed tomography-based fractional flow reserve with instantaneous wave free ratio to detect hemodynamically significant coronary stenosis. Clin Res Cardiol.

